# A Systematic *In Silico* Search for Target Similarity Identifies Several Approved Drugs with Potential Activity against the *Plasmodium falciparum* Apicoplast

**DOI:** 10.1371/journal.pone.0059288

**Published:** 2013-03-26

**Authors:** Nadlla Alves Bispo, Richard Culleton, Lourival Almeida Silva, Pedro Cravo

**Affiliations:** 1 Instituto de Patologia Tropical e Saúde Pública/Universidade Federal de Goiás/Goiânia, Brazil; 2 Malaria Unit/Institute of Tropical Medicine (NEKKEN)/Nagasaki University/Nagasaki, Japan; 3 Centro de Malária e Doenças Tropicais.LA/IHMT/Universidade Nova de Lisboa/Lisboa, Portugal; State University of Campinas, Brazil

## Abstract

Most of the drugs in use against *Plasmodium falciparum* share similar modes of action and, consequently, there is a need to identify alternative potential drug targets. Here, we focus on the apicoplast, a malarial plastid-like organelle of algal source which evolved through secondary endosymbiosis. We undertake a systematic *in silico* target-based identification approach for detecting drugs already approved for clinical use in humans that may be able to interfere with the *P. falciparum* apicoplast. The *P. falciparum* genome database GeneDB was used to compile a list of ≈600 proteins containing apicoplast signal peptides. Each of these proteins was treated as a potential drug target and its predicted sequence was used to interrogate three different freely available databases (Therapeutic Target Database, DrugBank and STITCH3.1) that provide synoptic data on drugs and their primary or putative drug targets. We were able to identify several drugs that are expected to interact with forty-seven (47) peptides predicted to be involved in the biology of the *P. falciparum* apicoplast. Fifteen (15) of these putative targets are predicted to have affinity to drugs that are already approved for clinical use but have never been evaluated against malaria parasites. We suggest that some of these drugs should be experimentally tested and/or serve as leads for engineering new antimalarials.

## Introduction

Malaria remains a serious public health problem in many tropical countries [1]. As there is still no effective vaccine available, treatment and prevention of the disease is primarily based on antimalarial drug administration and anti-vector measures, respectively. The efficacy of antimalarial drug treatment is compromised by the malaria parasite’s ability to develop drug-resistance, and by the dearth of new and effective antimalarials in the drug-design pipeline. There is, therefore, an urgent need for the discovery of new antimalarial drugs.

The main antimalarials presently approved for clinical use act mainly on two parasite metabolic pathways: haemoglobin degradation and nucleic acid synthesis. However, with the exception of artemisinin derivatives, parasite resistance has evolved and become common for the currently used antimalarial drugs. One of the underlying phenomena contributing to the emergence of drug resistance, is that resistance to different drugs is often controlled by similar molecular mechanisms and consequently the evolution of resistance to one particular compound may impact on the efficacy of others. For instance, resistance to quinine-derived drugs, such as mefloquine and lumefantrine, as well as to the structurally unrelated artemisinin derivatives, has been shown to be modulated by mutations and/or amplification of the Multidrug Resistance Protein homologue-1, PfMDR1 [2]. Similarly, resistance to drugs that block parasite nucleic acid synthesis, such as sulfadoxine, pyrimethamine and proguanil, is largely conferred by point mutations in genes encoding two enzymes, dihydrofolate reductase (DHFR) and the dihydropteroate synthase (DHPS) [3].

When considering the design of new antimalarial drugs, it is, therefore, imperative to investigate alternative antimalarial molecular targets. One such strategy has focused on the apicoplast, a non-photosynthetic malarial plastid which was first described in the 1990’s [4], [5] and recently confirmed to have been acquired by secondary endosymbiosis of a plastid-containing red alga [6]. The apicoplast’s genome is small (≈35 kb), and the organelle harbors several unique metabolic functions, mostly accomplished by proteins that are nuclear-encoded and later imported into its lumen [7]. These unique metabolic features represent an attractive starting point for therapeutic intervention, since they are mostly of plant/algal origin, a fact that may heighten the target selectivity of antimalarial drugs and/or reduce the probabilities of toxicity to humans. Importantly, previous studies have already confirmed that the apicoplast is vulnerable to drugs that affect its metabolic functions, such as replication, nucleic acid metabolism, translation, fatty acid synthesis and isoprenoid biosynthesis [8].

A conventional drug development strategy may involve both *de novo* drug discovery and the improvement of inhibitors of individually validated targets. Although this process represents an efficient strategy to develop novel antimalarial drugs, it is usually costly and time-consuming. An alternative and/or complementary approach is to screen existing clinically approved drugs for previously unidentified antimalarial activity, thus speeding up the discovery of new therapies. As such drugs are already approved for use in humans for other purposes, they can more easily enter human clinical efficacy trials under existing drug administration guidelines.

There are a number of different publicly available web-based databases which provide information on thousands of known therapeutic protein targets, the diseases that they are involved in, evidence for which pathways they play a role in, and the corresponding drugs which are directed at each of them. Using three of these databases, DrugBank [9], STITCH3.1 [10] and Therapeutic Target Database (TTD) [11], we adopted an *in silico* “top-down” approach to identify proteins localized to the *P. falciparum* apicoplast that may be targeted by drugs which are already in use in human clinical practice.

## Materials and Methods

### Compilation of a List of Apicoplast-targeted Proteins

A list of *Plasmodium falciparum* apicoplast-targeted proteins has been previously published by Ralph et al, 2004 [7]. The description of the methods that were used to identify proteins predicted to contain apicoplast-targeting sequences is detailed in that paper [7]. For the present work, this list was accessed and each protein entry was logged on to an Excel file datasheet. Proteins were grouped consecutively in a datasheet column depending on their predicted metabolic function and according to the classification available in the “Malaria Parasite Metabolic Pathways” web page [12]. Their identification codes (IDs) were then retrieved from the GeneDB *P. falciparum* genome database [13] and logged onto the corresponding column as a clickable hyperlink. We further checked the annotation of each single predicted peptide and corrected it, if necessary, according to the recent updated annotations of the GeneDB database. Next, we retrieved each individual predicted amino acid sequence and copied it to the corresponding column for each protein.

### Identification of Putative Drug Targets Using Publicly Available Drug Databases Overall Strategy

Each of the *P. falciparum* predicted protein sequences from the list compiled above was treated as a putative drug-target and consequently used to interrogate three different publicly available web databases that provide synoptic data on drugs and their primary or putative drug targets: DrugBank [9], STITCH3.1 [10] and the Therapeutic Target Database (TTD) [11]. Our search strategy for all three databases was based on the principle of “target similarity” whereby each query (*P. falciparum* apicoplast protein) is compared for similarity with all known drug targets contained within each of the databases. In cases where homologous drug targets were identified, all proteins with an output expectation value (E-value) lower than 1e−5 for DrugBank and TTD were listed as potential targets. In the case of STICH3.1, a score from 0 to 1.0 is given, rather than an expectation value. Thus, only proteins with a score above 0.7 were considered potential targets. We further filtered all positively identified targets through inclusion in the list of only those proteins that were indicated to interact with compounds that have already been approved for clinical use in humans.

### Database Commands for DrugBank [9]

Starting from the homepage, the option “search” → “sequence search” was chosen from the toolbar menu. The query protein sequence was then entered in FASTA format and the remaining default search parameters were used.

### Database Commands for STITCH3.1 [10]

Starting from the homepage, the option “protein sequence” from the menu box was clicked. The query protein sequence was then entered in FASTA format and the “Go” icon was clicked. When positive results were obtained only targets with a score above 0.7 were considered.

### Database Commands for Therapeutic Targets Database [11]

Starting from the homepage, the option “target similarity search” from the menu box was clicked. The query protein sequence was then entered in FASTA format and the “Search” icon was clicked. When positive results were obtained only targets with an Expectation value (E-value) score lower than 1e−5 were considered for further analyses.

### Compilation of the “Predicted Targets List”

After running each of the *P. falciparum* protein sequences in the three databases, all proteins with negative results (negative hits) were excluded from further analyses, whilst predicted targets from each database were compiled into a single Excel file, hereafter named “predicted targets list”. The following parameters associated with each positive hit were entered into the spreadsheet: “Homologous target(s) name(s) and target ID(s)” (DrugBank and TTD), “E-value(s) or score(s)” (DrugBank, STITCH3.1 and TTD), “Drug type(s)” (DrugBank, STITCH3.1 and TTD), “Drug name(s)” (DrugBank, STITCH3.1 and TTD), “Drug ID(s)” (DrugBank) and “Toxicity” (DrugBank).

Each positive hit was further cross-examined in the TDR targets Database [14] for its “druggability index”, which is an estimate of the likelihood of a protein being druggable and ranges from 0 to 1.0, with a value of ≈0.2 corresponding to average druggability. To do this, we clicked on the “targets” item in the web site’s menu and then ticked “*Plasmodium falciparum*” from the pathogen species list. We next filtered targets by entering each protein’s identifier (ID) in the corresponding box in the “Filter targets based on” search options. After clicking on the “search” icon, the above variable was retrieved from the target’s page and was subsequently recorded in the “predicted targets list” file.

### List of Drugs Yet to be Tested Against Malaria

Finally, we carried out a literature search using PubMED in order to identify approved drugs that have never been evaluated against malaria parasites by querying all drugs associated with each positive hit in the list. Our definition of “evaluation” embraces *in vitro* and/or *in vivo* testing and any malaria parasite species. Therefore if a given drug is noted as “not tested”, it means that no publication records were found after either of the following search details were entered in PubMED: 1. (“drug name”[MeSH Terms] OR “drug name”[All Fields]) AND (“plasmodium”[MeSH Terms] OR “plasmodium”[All Fields]) and 2. (“drug name”[MeSH Terms] OR “drug name”[All Fields]) AND (“malaria”[MeSH Terms] OR “malaria”[All Fields]), or that the study(ies) retrieved were insufficiently informative to infer the potential usefulness of the drug as an antimalarial.

## Results

### Schematic Summary

**Figure 1:**
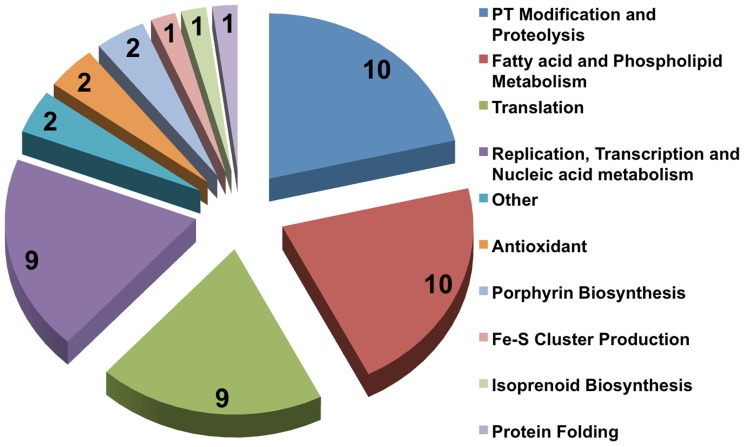
Flowchart summarizing the work pipeline and corresponding results. (*denotes the targets that were discarded on the basis of having chemical affinity to dietary supplements/nutraceuticals).

For ease of understanding, the overall results of this work are represented as a flow chart in Figure 1, the detailed results of which are given in the following sections.

### Compilation of the “Predicted Targets” List

Each of the *Plasmodium falciparum* proteins predicted to contain an apicoplast target signal was entered into a single Excel file as described in Materials and Methods. A list of a total 595 candidate target protein sequences was thus compiled and each was subsequently allocated either of the following predicted metabolic functions: “Replication, Transcription and Nucleic acid metabolism”, “Translation”, “Fatty acid and Phospholipid Metabolism”, “Transport”, “Antioxidant”, “Protein Folding”, “Fe-S Cluster Production”, “Porphyrin Biosynthesis”, “Post-translational Modification and Proteolysis” and “Other/unknown function” (Table S1). Each of these protein sequences in the list was interrogated for target similarity in the three databases used (DrugBank, STITCH3.1 and TTD), producing a list of a total seventy-two (72) “positive hits” (≈12% of the total predicted apicoplast peptides) (Table S2). We decided to use all three databanks because each of them may contain different drug-target datasets and, consequently, the probability of targets being missed due to insufficient screening is reduced. Indeed, no single database was capable of identifying all 72 predicted targets: DrugBank, STICH3.1 and TTD identified exclusively 45, 8 and 11 predicted targets, whilst the remaining 8 targets were identified by two or three of the databases. Detailed information about the predicted targets and their associated compounds is provided in Table S2.

**Figure 2:**
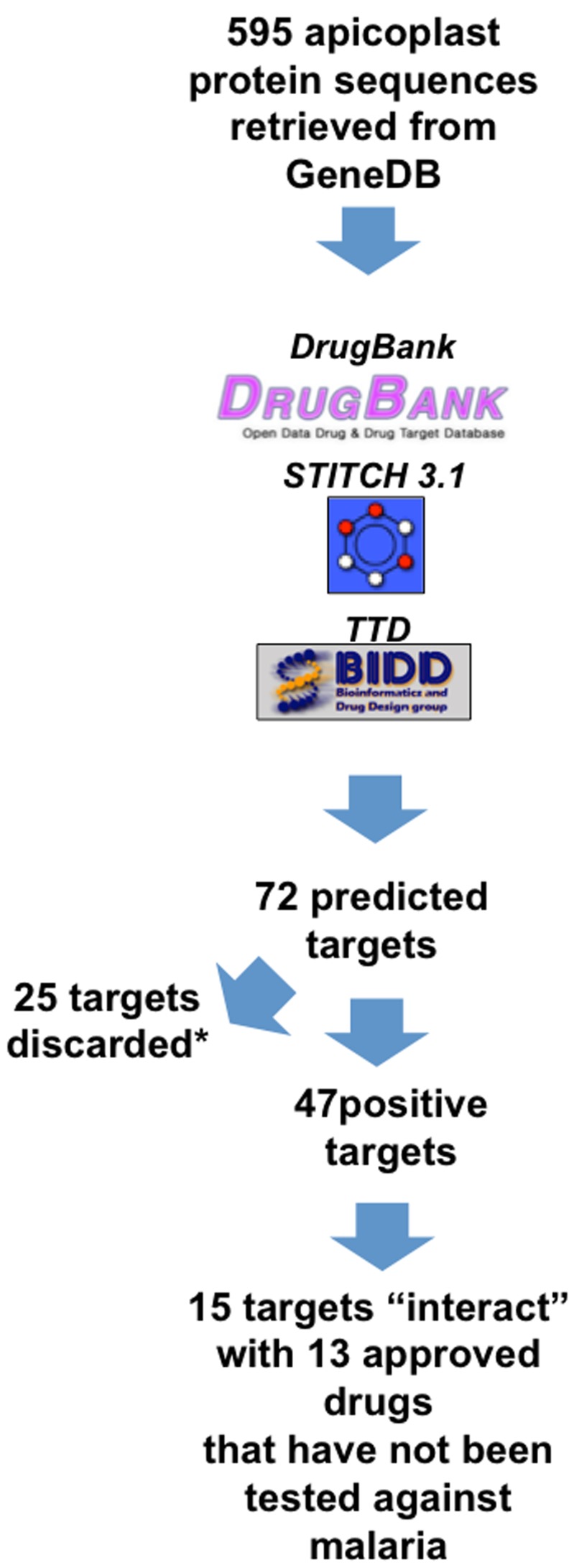
Distribution of the expected apicoplast targets according to their predicted metabolic function in the apicoplast.

Approximately one third of the positively identified targets (N = 25) were predicted to react with compounds belonging to the “Dietary supplement/nutraceutical” class. Since these compounds are unlikely to exhibit antimalarial activity, these targets and their associated compounds were excluded from further analyses. The distribution of the remainder 47 potential targets according to their predicted metabolic function is depicted in Figure 2. Eighty percent (80%, N = 38) of these 47 positive hits are distributed between four main metabolic function groups: “Replication, Transcription and Nucleic acid metabolism”, “Translation”, “Fatty acid and Phospholipid Metabolism” and “Post-translational Modification and Proteolysis”.

### Previously Untested Drugs

In order to evaluate which of the drugs associated with the predicted targets had been tested against malaria parasites and which had not, we ran a literature search in PubMed as described above. Out of a total of 47 positive hits from the list compiled above, 32 targets were associated with drugs whose activity has been previously evaluated against malaria parasites. Examples of some of these drugs and their corresponding targets are given in Table 1. However, for 15 of the predicted targets, there were a total of 13 corresponding drugs that have never been experimentally or clinically tested against malaria or whose evaluation required further studies (Table 2). The metabolic functions predicted to be targeted by each of these drugs in the apicoplast are the following: Replication, Transcription and Nucleic acid metabolism (5 drugs: azelaic acid, lucanthone, bleomycin, rifabutin and gemcitabine), Fatty acid and Phospholipid Metabolism (3 drugs: ethionamide, nitrofurazone and isoxyl) and post-translational modification and proteolysis (5 drugs: nitroxoline, gallium nitrate, sulcrafate, remikeren and aliskeren). Two of these drugs (azelaic acid and sulcrafate) are predicted to interfere with more than one peptide and one particular predicted target (plasmepsin X) is expected to have affinity to more than one drug. The list of these drugs, their targets, associated toxicity (if any) and each target’s druggability index is depicted in Table 2.

**Table 1:** Examples of drug-target associations previously determined, that were correctly identified in the present study.

Drug(s)	*P. falciparum* target(s) ID	Identity to (E-value or score)	Pathway	Reference(s)
Several quinolones	DNA GyrAse a-subunit, putative PF3D7_1223300	DNA gyrase subunit A (P72524 - GYRA_STRPN) (1e−50)	Replication	[8]
Fusidic acid	elongation factor G, putativePF3D7_0602400	Elongation factor G (P13551 - EFG_THETH) (8.7e−75)	Translation	[17]
Tetracycline	apicoplast ribosomal protein S14p/S29eprecursor, putative PF3D7_1137500	30S ribosomal protein S14 (P0AG59 - RS14_ECOLI) (7.5e−15)	Translation	[32]
Fosmidomycin	1-deoxy-D-xylulose 5-phosphatereductoisomerase PF3D7_1467300	0.99	Isoprenoid biosynthesis	[16]
Triclosan	enoyl-acyl carrier reductase PF3D7_0615100	0.99	Fatty acid synthesis	[8]
Geldanamycin	heat shock protein 90, putative	0.80	Protein folding	[33]
Halofantrine	plasmepsin VIII PF3D7_1465700	Plasmepsin-2 (P46925 - PLM2_PLAFA) (9e−23)	Proteolysis	[31]

(codes in brackets represent the target Identity Code of DrugBank. In the cases of Fosmidomycin, Triclosan and Geldanamycin, there are no homologous targets represented because they were identified using STITCH3.1 which uses an algorithm where homologous targets are not displayed).

**Table 2:** New drug-target associations disclosed in the present study.

Drug (brand names)	Drug category(ies)	Toxicity	*P. falciparum* target(s) ID	Identity to (E-value)		TDRT Druggability	Metabolic Function
**Azelaic acid** (Azelex, Emerox 1110, Emerox 1144, Emery's L110, Finacea, Finevin, Skinoren)	Antineoplastic Agents Dermatologic Agents	Oral LD50 in rat: >5 g/kg	5′-3′ exonuclease, N-terminal resolvase-like domain, putative PF3D7_0203900		DNA polymerase I (P00582 - DPO1_ECOLI) (2e−12)	NA	DNA repair
			plastid replication-repair enzyme (PREX) PF3D7_1411400		DNA polymerase I (P00582 - DPO1_ECOLI) (4e−50)	NA	DNA repair
**Lucanthone** (Miracil D, Miracol, Nilodin, Scapuren, Tixantone)	Radiation-Sensitizing Agents Anticancer AgentsSchistosomicides	NA	AP endonuclease (DNA-(apurinicor apyrimidinic site) lyase),putative PF3D7_0305600		DNA-(apurinic or apyrimidinic site) lyase (P27695 - APEX1_HUMAN) (8e−24)	NA	DNA repair
**Bleomycin** (Blenoxane, Bleo)	Antimetabolites Antibiotics, Antineoplastic	Excessive exposure may cause fever, chills, nausea, vomiting, mental, confusion, and wheezing. Bleomycin may cause irritation to eyes, skin and respiratory tract.It may also cause a darkening or thickening of the skin. It may cause an allergic reaction.	DNA ligase 1 PF3D7_1304100		DNA ligase 1 (P18858 - DNLI1_HUMAN) (4e−118)	NA	Replication
**Rifabutin** (Alfacid, Ansamycin, Mycobutin)	Anti-Bacterial Agents Antibiotics, Antitubercular	LD50 = 4.8 g/kg (mouse, male)	DNA-directed RNA polymerase alpha chain, putative PF3D7_1307600		DNA-directed RNA polymerase alpha chain(P0A7Z4 - RPOA_ECOLI) (1e−8)	NA	Replication
**Gemcitabine** (DDFC, DFDC, Gemcin) Gemcitabine hydrochloride, Gemtro, Gemzar, GEO)	Antineoplastic Agents Antiviral Agents Radiation-Sensitizing Agents Antimetabolites Enzyme Inhibitors Immunosuppressive Agents	Myelosuppression, paresthesias, and severe rash were the principaltoxicities, LD50 = 500 mg/kg(orally in mice and rats)	UMP-CMP kinase, putative PF3D7_0111500		UMP-CMP kinase (P30085 - KCY_HUMAN) (9e−22)	NA	Nucleic acid metabolism
**Ethionamide** (Aethionamidum, Aetina, Aetiva, Amidazin, Amidazine, Atina, Bayer 5312, Ethimide, Ethina, Etimid)	Leprostatic Agents Antitubercular Agents Fatty Acid Synthesis Inhibitors	Symptoms of overdose include convulsions, nausea, and vomiting	enoyl-acyl carrier reductase PF3D7_0615100		Enoyl-[acyl-carrier-protein] reductase [NADH] (P0A5Y6 - INHA_MYCTU) (4e−9)	NA	FAS
**Nitrofurazone** (Actin-N, Aldomycin, Alfucin, Amifur, Babrocid, Becafurazone, Biofuracina, Biofurea, Chemofuran, Chixin)	Anti-Infective Agents Anti-Infective Agents, Local Trypanocidal Agents Anti-Infective Agents, Urinary	Rat LD50 = 590 mg/kg; Allergic contact dermatitis is the most frequently reported adverse effect, occurring in approximately 1% of patients treated.	lipoamide dehydrogenase,putative PF3D7_0815900		Glutathione reductase (P06715 - GSHR_ECOLI) (1e−18)	0.3	FASAntioxidant
**Isoxyl** (Thiocarlide)	Anti-bacterial agents	NA	stearoyl-CoA delta 9 desaturase, putative PF3D7_0511200		Acyl-CoA desaturase (TTDS00516) (2e−52)	0.1	FAS
**Nitroxoline** (Galinok, Isinok, Nicene forte, Noxibiol, Noxin)	Antifungal AgentsAnti-Infective Agents,Urinary	NA	methionine aminopeptidase 1c, putative PF3D7_0804400		Methionine aminopeptidase 1 (P53582 - AMPM1_HUMAN)(2e−29)	0.3	Proteolysis
**Gallium Nitrate** (Ganite)	Antineoplastic Agents	NA	protein phosphatase, putative PF3D7_1469200		Protein-tyrosine-phosphatase (Q9S427–Q9S427_9GAMM)(2e−16)	NA	Phosphorylation
**Sucralfate** (Antepsin, Apo-sucralfate, Carafate, Sucramal, Sulcrate, Sulcrate Suspension Plus, Ulcar, Ulcerban, Ulcerlmin, Ulcermin)	Anti-Ulcer Agents	Acute oral toxicity (LD50) in mice is >8000 mg/kg. There is limited experience in humans with overdosage of sucralfate. Sucralfate is only minimally absorbed from the gastrointestinal tract and thus risks associated with acute overdosage should be minimal.	plasmepsin X PF3D7_0808200		Pepsin A (P00790 - PEPA_HUMAN) (7e−43)	0.6	Proteolysis
			plasmepsin I (PMI) PF3D7_1407900		Pepsin A (P00790 - PEPA_HUMAN) (5e−41)	0.8	Proteolysis
			plasmepsin III,histo-aspartic protease (HAP) PF3D7_1408100		Pepsin A (P00790 - PEPA_HUMAN) (2e−34)	0.8	Proteolysis
			plasmepsin VII PF3D7_1033800		Pepsin A (P00790 - PEPA_HUMAN) (2e−25)	0.3	Proteolysis
**Remikiren**	Antihypertensive Agents Protease Inhibitors	NA	plasmepsin X PF3D7_0808200		Renin (P00797 - RENI_HUMAN) (1e−26)	0.6	Proteolysis
**Aliskiren** (Rasilez, Tekturna)	Antihypertensive Agents	The most likely manifestation of overdosage would be hypotension	plasmepsin X PF3D7_0808200		Renin (P00797 - RENI_HUMAN) (1e−26)	0.6	Proteolysis

(NA: not available; codes in brackets represent the target Identity Code of DrugBank. Toxicity data is cited from DrugBank; FAS: Fatty Acid Synthesis; LD50: drug dose that results in death of 50% of the animals).

## Discussion

The main objective of this work was to identify drugs that have been approved for clinical use in humans for conditions other than malaria, which may have the potential to interfere with the function of the apicoplast. In validation of our approach, all the main drugs previously shown to target the apicoplast and their known targets were identified by our methodology (Table S2, Table 1) and the following illustrative examples are given. The antibiotics ciprofloxacin and doxycycline and their respective targets, the apicoplast’s DNA gyrase and small ribosomal subunits [8], were correctly pinpointed by our approach. Fosmidomycin, a drug that is known to target the apicoplast’s 1-deoxy-D-xylulose 5-phosphate reductoisomerase (DOXP) [15], [16], was appropriately identified. Fusidic acid and its likely target, elongation factor G [17], involved in the process of translation, were correctly pinpointed and associated. Consequently, we were confident that our overall strategy for identifying anti-apicoplast drugs is valid. Following this precondition, we were able to identify thirteen drugs that have not yet been evaluated against malaria parasites. These drugs are supposed to inhibit targets that are involved in metabolic functions of the apicoplast that have been shown to render the parasite vulnerable to drugs [8]. For this reason we suggest that the antimalarial activity of at least some of these compounds should be investigated further. In the ensuing paragraphs we refer specifically to five of these drugs that we suggest may be good candidates for antimalarial testing, highlighting their advantages but also the constraints that may limit their direct use *in vivo*.

Azelaic acid (AA) has been shown to interfere with DNA synthesis in bacteria [18] and its oral toxicity in mice appears to be low (>5 g/kg, data from DrugBank). In the present work, our search suggests that AA may be able to interfere with two targets involved in DNA repair within the *P. falciparum* apicoplast: a peptide with a 5′-3′ exonuclease, N-terminal resolvase domain (PF3D7_0203900) and a plastid replication-repair enzyme (PREX) (PF3D7_1411400). Additionally, according to the data available in the DrugBank database, AA has a log *P* value of 1.7, indicating that the drug may diffuse well through biomembranes [8]. Although this property is not a pre-requisite for the success of drugs targeting the apicoplast’s biology [8], it may come as a benefit for apicoplast-targeting drugs, which have to cross a total of six membranes. However, AA is used commercially in the form of a topically applied cream and thus the practical aspects of testing it as an antimalarial may present some challenges.

Lucanthone is one of the earliest described schistosomicides [19] and was predicted to target the apicoplast’s putative AP-endonuclease (PF3D7_0305600) in the present work. It was later replaced largely by hycanthone, its active metabolite. Although there are no records in the literature about the evaluation of lucanthone’s antimalarial activity, hycanthone has been hypothesized to possess antimalarial activity in a virtual screen against *P. falciparum* with an IC50 value below 5 microM [20]. In the present day lucanthone is used as an anti-cancer agent where it has been shown to be well tolerated by humans with no hematological or gastro-intestinal toxicity at clinically tolerated doses (data from DrugBank). However, this contrasts with earlier suggestions where in past shistosomiasis treatment with lucanthone was reported to produce side effects such hepatotoxicity and gastrointestinal disturbances following intramuscular injection [21].

Isoxyl (Thiocarlide), a thiourea derivative that was used successfully for the clinical treatment of TB during the 1960s, has been shown to display significant antimycobacterial activity *in vitro* and is effective against multi-drug resistant strains of *Mycobacterium tuberculosis* [22]. In Mycobacteria, isoxyl (ISO) has been shown to inhibit the synthesis of oleic acid and this effect is directly attributable to the inhibitory effect of the drug on the membrane-associated stearoyl-coenzyme A (CoA) (Δ9) desaturase DesA3 (Rv3229c) [23]. Results from the present work suggest that isoxyl may also be able to inhibit the *P. falciparum* apicoplast homologue (E-value = 2e−52), a putative stearoyl-CoA delta 9 desaturase (PF3D7_0511200). Also, ISO has no known side-effects [24], which makes it highly appropriate for clinical use in humans. One likely downside of ISO is the fact that it has a high log*P* value of ≈5.8 (http://www.chemspider.com/Chemical-Structure.2272774.html), which makes it virtually insoluble in water with consequent poor dissolution and bioavailability when it is delivered exclusively by the oral route [24].

Nitroxoline (synonym 5-Nitroxin) is an active urinary antibacterial agent which has been used since 1962 against susceptible gram-positive and gram-negative organisms commonly found in urinary tract infections [25]. It has been suggested that its antibacterial activity may stem from the metal ion complexation vital for bacterial growth [26]. More recently, it was discovered that that nitroxoline has antiangiogenic properties, which also makes it useful as an anti-cancer drug [27]. We found that Nitroxoline may be able to interfere with the apicoplast-targeted *P. falciparum* putative methionine aminopeptidase 1c (PF3D7_0804400), which is homologous to the human enzyme and is expected to be involved in proteolysis within the apicoplast. Interestingly, nitroxiline was tested in early *in vitro* studies where it was shown to display exceptional activity against *P. falciparum*, exhibiting an ED50 of approximately 63 nM at 48h post-exposure, a value which reflected roughly 10X higher potency than quinine sulfate in that same study, under identical conditions [28]. In addition to what appears to be a level of high potency *in vitro*, nitroxoline displays a *logP* of 1.9 (data from DrugBank), which should favor its ability to cross the membranes required to reach its target. Curiously, the assessment of nitroxoline as a potential antimalarial for clinical use *in vivo* appears to have never been followed-up. For all reasons cited, we argue here that nitroxoline should be prioritized for further evaluations of its potential value as an antimalarial drug.

Lastly, we refer to sulcrafate, possibly one of the least obvious drugs to hold antimalarial activity, due to the fact that it is not an anti-infective agent per se, but rather an anti-ulcer compound. Sulcrafate is an approved small molecule, which is a basic aluminum complex of sulfated sucrose and is orally employed for prevention and treatment of several gastrointestinal diseases including gastroesophageal reflux, gastric and duodenal ulcer [29]. Sucralfate acts, at least partially, through inhibition of the human pepsin A enzyme [30], which was shown here to be homologous to four *P. falciparum* plasmepsins localized to the apicoplast (plasmepsin X: PF3D7_0808200, plasmepsin I (PMI): PF3D7_1407900, plasmepsin III,histo-aspartic protease (HAP): PF3D7_1408100 and plasmepsin VII: PF3D7_1033800). Interestingly, halofantrine, a well-known highly effective antimalarial has been recently suggested to inhibit plasmepsin X, one of sulcrafate’s predicted targets [31]. Due to its own therapeutic nature, sulcrafate is only minimally absorbed from the gastrointestinal tract (data from DrugBank) and consequently it is unlikely to reach systemic therapeutic levels in patients treated orally. However, since sulcrafate is commercially available in powder form, it may be tested directly after dilution in an appropriate vehicle in experimental *in vivo* models of malaria with administration via a route other than oral, such as intra-venous or intra-peritoneal.

Besides the drugs highlighted above, eight further drugs are predicted to be capable of interfering with apicoplast targets (Table 2). They were not discussed in detail because we envisage that some of their inherent properties may render them less suitable than the above as antimalarial agents. For instance, bleomycin, gemcitabine and gallium nitrate are three anti-neoplastic agents and for this reason are more likely to cause severe side-effects/toxicity in humans in case their direct use is considered. Indeed, although gallium nitrate’s toxicity parameters are not available, considerable toxicity is expected from bleomycin and gemcitabine (Table 2). In other cases, such as those of rifabutin and ethionamide, we considered that the output expectation value (E-value) for target homology insufficiently significant to infer the target’s prediction with a high degree of confidence. Remikeren and aliskeren are two antihypertensive agents and for that reason, should present increased challenges as to their short-term applicability as antimalarials.

### Conclusions

We describe a systematic *in silico* approach to identify drugs that have been clinically approved for human use, but have never been evaluated against malaria parasites based on the principle of “homologous drug target screening”. In doing so, we were able to identify thirteen such drugs that we suggest justify evaluation as antimalarials. We stress the fact that we have no experimental evidence to suggest that any of the newly identified drugs will either display antimalarial activity and/or affect the suggested targets. It also is possible that their *in viv*o potencies may be compromised by absorption, distribution, metabolism, excretion ad toxicity (ADMET) properties or by lack of appropriate chemical affinity with their putative target(s). Nevertheless, primary *in vitro* drug screens may provide insights into their ability to inhibit parasite growth and, if any promising activities are disclosed, they could constitute important leads to the discovery of novel antimalarials.

## Supporting Information

Table S1 **List of apicoplast-targeted proteins with predicted aminoacid (AA) sequences.**
(XLSX)

Table S2 **Predicted targets list.** (DB: DrugBank; TTD: Therapeutic Targets Database: DS: dietary supplement or nutraceutical. TDRT: Tropical Disease Research Targets; Blank cells denote that no data was available).
(XLSX)
