# Correction: A Systematic *In Silico* Search for Target Similarity Identifies Several Approved Drugs with Potential Activity against the *Plasmodium falciparum* Apicoplast

**DOI:** 10.1371/annotation/0bbd3579-5212-4dcf-a5ef-dd3d8e26f287

**Published:** 2013-06-28

**Authors:** Nadlla Alves Bispo, Richard Culleton, Lourival Almeida Silva, Pedro Cravo

Figures 1 and 2 were incorrectly switched. 

Figure 1 should appear with the title and legend of Figure 2, and Figure 2 should appear with the title and legend of Figure 1. The titles and legends of the figures are in correct order. 

